# When Speech Stops, Gesture Stops: Evidence From Developmental and Crosslinguistic Comparisons

**DOI:** 10.3389/fpsyg.2018.00879

**Published:** 2018-06-01

**Authors:** Maria Graziano, Marianne Gullberg

**Affiliations:** ^1^Lund University Humanities Lab, Lund University, Lund, Sweden; ^2^Centre for Languages and Literature, Lund University, Lund, Sweden

**Keywords:** gesture, speech production, language development, second language acquisition, crossmodal coordination

## Abstract

There is plenty of evidence that speech and gesture form a tightly integrated system, as reflected in parallelisms in language production, comprehension, and development (McNeill, 1992; Kendon, 2004). Yet, it is a common assumption that speakers use gestures to compensate for their expressive difficulties, a notion found in developmental studies of both first and second language acquisition, and in theoretical proposals concerning the gesture-speech relationship. If gestures are compensatory, they should mainly occur in disfluent stretches of speech. However, the evidence is sparse and conflicting. This study extends previous studies and tests the putative compensatory role of gestures by comparing the gestural behavior in fluent vs. disfluent stretches of narratives by competent speakers in two languages (Dutch and Italian), and by language learners (children and adult L2 learners). The results reveal that (1) in all groups speakers overwhelmingly produce gestures during fluent speech and only rarely during disfluencies. However, L2 learners are significantly more likely to gesture in disfluency than the other groups; (2) in all groups gestures during disfluencies tend to be holds; (3) in all groups the rare gestures completed in disfluencies have both referential and pragmatic functions. Overall, the data strongly suggest that when speech stops, so does gesture. The findings constitute an important challenge to both gesture and language acquisition theories assuming a mainly (lexical) compensatory role for (referential) gestures. Instead, the results provide strong support for the notion that speech and gestures form an integrated system.

## Introduction

In a seminal paper entitled *So you think gestures are non-verbal?* David McNeill challenged the then dominant view of gestures as a communicative frill of no consequence to our understanding of language and linguistic processing (McNeill, 1985). The paper listed arguments for why gestures are in fact verbal (i.e., linguistic), by highlighting their close relationship with spoken language in language development, in language break-down, and in language processing. He argued that speech and gesture develop in parallel in childhood, that the modalities break down together, and that they are processed in parallel in crossmodal information integration. There is now a substantial literature to support this view providing both behavioral and neurocognitive empirical evidence to show that speech and gesture form an integrated mode of expression in production and comprehension (e.g., Kendon, 1980, 2004; McNeill, 1992, 2005; Willems and Hagoort, 2007 for overviews), in development (e.g., Capirci and Volterra, 2008; Colletta et al., 2015 for overviews), and across different spoken languages (Kita, 2009 for an overview). Yet, despite the evidence for such crossmodal integration, both empirical studies and theoretical proposals concerning the speech-gesture relationship often see gestures as having mainly a facilitating or compensatory function, helping speakers to overcome expressive difficulties (Gullberg, 1998, 2011 for overviews). However, the evidence concerning the precise link between speech break-down or disfluency and gestures remains contradictory. Therefore, the current study aims to examine the distribution of gestures relative to disfluencies in competent adult native speakers of two languages, and of language learners, both children and adults, in order to shed some light on the putative compensatory role of manual gestures, extending previous studies. In the following, we review the empirical and theoretical background to the study of disfluency in general, and to the temporal and functional relationship between speech and gesture specifically, including possible crosslinguistic differences, before turning to the current empirical study.

## Background

Despite ever-growing evidence for the integrated nature of speech and gesture, many empirical studies still view gestures as serving mainly a compensatory function. For example, in many studies of infants or very young children, gestures are described as behaviors preceding and preparing for language (Bates, 1979; Volterra et al., 1979; Liszkowski, 2008), paving the way for and predicting later linguistic development (e.g., Morford and Goldin-Meadow, 1992; Iverson et al., 1994; Capirci et al., 1996, 2005; Butcher and Goldin-Meadow, 2000; Özçalişkan and Goldin-Meadow, 2005; Pizzuto et al., 2005), and even facilitating access to the child lexicon (e.g., Pine et al., 2007). Gestures are thus generally implicitly described as having a facilitating function. In contrast, in adult second language acquisition and bilingualism studies, the compensatory view is explicit. Adult learners are often observed to be producing more gestures when speaking their second compared to their first language. This behavior is generally described as reflecting a compensatory effort to overcome lack of skill and fluency in the weaker language (Gullberg, 1998, 2011), or even as activating items in the mental lexicon (e.g., Nicoladis et al., 2007, 2009). Finally, studies of atypically developing or impaired populations also display a compensatory view of gesture. Children with Specific Language Impairment (SLI) or with Down syndrome show higher gesture rates than typically developing peers (e.g., Fex and Månsson, 1998; Stefanini et al., 2008), and so do aphasic patients, especially those with word retrieval impairments (e.g., Feyereisen, 1983; Hadar et al., 1998; Rose, 2006 for an overview). These higher gesture rates are all seen as evidence that gestures facilitate speaking or at least communicating.

Moreover, several theoretical accounts concerning the speech-gesture relationship also have compensatory foundations, revolving around how mainly referential^1^ gestures, which convey information about referents’ size, shape, movement or location, help speaking and thinking. For example, the Information Packaging Hypothesis (e.g., Alibali et al., 2000; Kita, 2000) suggests that referential gestures facilitate the conceptual planning of the spoken message, particularly of spatio-motoric concepts.^2^ A recent expanded version, the Gesture-for-Conceptualization Hypothesis (Kita et al., 2017), proposes that speakers can activate, manipulate, package, and explore spatio-motoric information both for speaking and thinking through referential gestures. Although there is an underlying strand of compensatory thinking in these theories, their scope is very broad and the notion of compensation is not explicit. In contrast, the Lexical Retrieval Hypothesis (Krauss and Hadar, 1999; Krauss et al., 2000; Morsella and Krauss, 2005) is explicitly compensatory, suggesting that the main role of referential gestures is to facilitate lexical retrieval from the mental lexicon through crossmodal priming. In studies testing this theory, participants are often asked to name objects, or to provide words to a given definition, and in some cases are put in a tip-of-tongue state. These studies find that speakers produce more referential gestures when they speak about spatial content, and crucially, when they are searching for a word that is difficult to retrieve or that is unfamiliar (Butterworth and Hadar, 1989; Morrel-Samuels and Krauss, 1992; Rauscher et al., 1996; Krauss, 1998; Morsella and Krauss, 2005). More specifically, the claim is that word retrieval is more successful when participants gesture during the word search, that is, during the disfluency. Under the argument that gestures facilitate word retrieval, the temporal link between gesture production and disfluencies becomes crucial.

### Disfluency and the Temporal Speech-Gesture Relationship

The vast literature on speech errors and disfluency in speech production has examined when and where in an utterance speakers interrupt speech (e.g., Maclay and Osgood, 1959; Goldman-Eisler, 1968; Hawkins, 1972; Beattie and Butterworth, 1979; Levelt, 1983, 1989; Clark, 1996 inter multa alia). They reveal that the beginning of a clause is a vulnerable site and that disfluencies also often occur before content words. In addition, these studies have also provided taxonomies of different types of disfluency markers (e.g., filled and unfilled pauses, interruptions, repetition, and lengthening). Studies have also shown that speakers prefer to self-correct (Schegloff et al., 1977), and favor fluency over accuracy in interaction, which means that they tend to interrupt speech not when the problem in encoding is detected, but rather when speakers are ready to produce a repair (Seyfeddinipur et al., 2008). Other studies indicate that filled pauses may have a signaling function much like discourse markers (Clark and Fox Tree, 2002), and that both forms and distribution of such filled pauses are language-specific (e.g., Trofimovich and Baker, 2006; de Leeuw, 2007). In adult L2 learners, (dis-)fluency is discussed in terms of proficiency and (foreign) language skills (e.g., Poulisse, 1999; Schmid and Fägersten, 2010; De Jong et al., 2013; Bergmann et al., 2015).

Studies that specifically examine gesture production in relation to disfluency draw on some of these findings. Most studies investigate the temporal relationship between the gestural movement and disfluency markers. They present contradictory evidence both regarding the exact timing of the gesture relative to the disfluency, and the presence/absence of gesture. For example, Butterworth and Beattie (1978) found that gestures were as likely to begin during a silent pause as during speech. Ragsdale and Silvia (1982) instead reported that gestures could begin just before or simultaneously with non-fluent speech. However, in this study a wide range of movements was included (posture change, body shifts, foot, leg, head, and hand movements), making assessments specifically for manual gestures difficult. Generally, these early studies suggest that gestures tend to occur in the neighborhood of disfluencies. However, later studies have instead reported that speech and gesture stop at the same time. For instance, it has been shown that in stuttering populations the two modalities are interrupted together (Mayberry et al., 1998; Mayberry and Jaques, 2000). In other studies gestures are shown to stop even before speech stops (Seyfeddinipur and Kita, 2001; Seyfeddinipur, 2006), or to be totally absent during pauses and other disfluency phenomena (Christenfeld et al., 1991; Yasinnik et al., 2005). Further to this, there is some evidence that in adult L2 speakers’ gestures are less frequent during disfluent than fluent speech (Gullberg, 1998). The evidence for how gestures and disfluency may be linked is thus mixed.

The explanations for the contradictory findings are likely to be methodological in nature. An obvious issue is that studies have focused on different kinds of movement involving various body parts (head, hands, feet, etc.), or manual gestures with particular functions such as referential gestures only versus looking at all gestural movements. This makes it difficult to assess comparability. Similarly, it is not always clear what kind of disfluency is involved (unfilled pauses only, or also filled pauses, repetitions, etc.). And most importantly, it is often unclear which part of the gestural movement is considered when the timing of a spoken disfluency and a gesture is compared: the whole gesture phrase (starting from the preparation and including the stroke and any post-stroke hold), or only the stroke/core movement phase, etc. (cf. Kendon, 1980, 2004). Claims about whether speech or gesture stops first, for example, must be very specific with regard to gesture phase or movement analyses (e.g., Seyfeddinipur and Kita, 2001; Seyfeddinipur, 2006). When more detail is provided, some studies find, for example, that it is specifically gesture holds (i.e., the momentary suspension of a movement en route) that tend to coincide with speech pauses (Yasinnik et al., 2005; Park-Doob, 2010), even in children aged nine (Esposito and Marinaro, 2007).

### Disfluency and Gestural Function

In addition to timing, studies present mixed evidence concerning what gestural functions occur in disfluencies. As indicated, the theories and many studies have focused on referential gestures expressing referential content in disfluency. However, some of the earlier studies indicated the presence of different gestural functions by referring to ‘break-down’ gestures (Beattie and Butterworth, 1979 following Freedman, 1972). McNeill (1985, 1992) have subsequently labeled these ‘butterworths’ or ‘conduit gestures’, highlighting how gestures in break-downs often refer to the break-down itself, not to the content of speech. Gullberg (1998, 2011) has provided empirical support for this view, showing that if native and second language speakers gesture during disfluencies, they often produce gestures that comment on the breakdown itself but do not represent the referential content of the sought words. Many of these gestures involve continued wrist turning to expose palms (labeled meta-pragmatic, or ‘thinking gestures’ by Gullberg, ‘cyclic gestures’ by Ladewig, 2014), or palm up gestures directed toward the interlocutor. Kendon (2004) calls many of these gestures that do not express referential content for pragmatic gestures. On the whole, however, evidence for what functions gestures have in disfluency is scarce.

### Disfluency and Crosslinguistic Comparisons

Relatedly, most studies concerned with gesture and disfluency are based on English production (except Italian in Esposito and Marinaro, 2007, and German in Seyfeddinipur and Kita, 2001; Seyfeddinipur, 2006). There are no direct crosslinguistic comparisons of the relationship between gesture and speech in disfluency. However, reports are found in the literature of differences in the distribution of gesture functions in speakers of different languages. For example, in a pioneering study Efron (1941/1972) observed that Italian immigrants in the United States produced more referential gestures than Yiddish-speaking immigrants, who instead tended to produce more pragmatic gestures. Similarly, Kendon (2004) observed a wider range of pragmatic gestures in Italian speakers than in British and American English speakers. Gullberg (1998) also observed that native Swedish speakers produced more referential gestures than native French speakers who instead produced more non-referential gestures (specifically beats). If gesture functions in disfluencies vary, then crosslinguistic preferences for referential or pragmatic gestures may interact with the kind of gestural behavior found in disfluency. However, gestures and disfluency has not been examined crosslinguistically, to our knowledge.

### Intermediate Summary

In sum, previous studies provide inconsistent evidence on the precise temporal relationship between gestures and (dis-)fluency, presumably due to methodological differences. This in turn makes it difficult to assess theoretical proposals such as the compensatory Lexical Retrieval Hypothesis in contrast to the view of speech and gesture as an integrated system. Moreover, there is only scant evidence for how gestures are functionally distributed during disfluent speech despite the latent relevance of gesture function to the theories about gesture and speech break-down. Further to this, direct crosslinguistic comparisons of speech disfluency and gesture are absent in the literature in spite of the potential importance of such comparisons for theoretical claims. Finally, data on language learners is scarce, looking specifically at disfluency rather than on general linguistic development in connection to gesture production. Therefore, to improve our understanding of whether speech and gestures form an integrated mode of expression or whether gestures mainly serve a compensatory or facilitating role in speech production, the current study aims to test the core predictions from the Lexical Retrieval Hypothesis, and examine the precise temporal and functional relationship between gestures and disfluencies in competent adult native speakers of two languages, and in language learners, children and adults.

## Current Study

The Lexical Retrieval Hypothesis predicts that (a) ongoing gestures should occur in stretches of *disfluent* compared to fluent speech if they are to help crossmodally prime lexical items; (b) that these gestures should have referential functions linking the gesture to the referential content of the lexical item sought. Further, assuming that language learners are more disfluent than competent speakers, we infer that the hypothesis would predict (c) that this state of affairs should hold especially for language learners. In contrast, the view of speech and gesture as an integrated system predicts that ongoing gestures should mainly occur in stretches of *fluent* speech compared to disfluent speech. It makes no predictions about gestural functions; however, previous observations suggest that ongoing strokes in disfluency may have a pragmatic rather than a referential function, commenting on the breakdown rather than reflecting the referential content of the sought lexical item. Finally, it predicts no differences between competent speakers and learners. Neither view makes predictions about crosslinguistic differences.

The current study addresses these issues and extends previous studies by comparing the gestural behavior during fluent and disfluent speech in (a) adult native speakers of Dutch vs. Italian; (b) child learners vs. adult competent speakers of Italian; and (c) adult Dutch second language learners of French vs. adult native Dutch speakers. We ask (1) whether speakers predominantly produce gestures with fluent or with disfluent speech; (2) whether gestures occurring with disfluencies tend to be ongoing strokes or holds; (3) whether ongoing strokes during disfluencies have referential or pragmatic functions; (4) and whether there are crosslinguistic differences between Dutch and Italian speakers.

### Method

#### Participants

The analyses draw on four multimodal corpora consisting of narrative production (story retellings) in a dyadic, interactive setting. The corpora are based on the narratives of 66 participants divided over four groups (cf. **Table 1**): children learning Italian aged four, six, and nine (*n* = 3 × 11, 22 female); adult Italian native speakers (*n* = 11, 7 female); adult Dutch native speakers (*n* = 11, 9 female), who are also second language learners of French (*n* = 11, 9 female). The corpora thus consist of adult native speakers of two languages (Dutch, Italian) allowing for a crosslinguistic comparison of ‘competent’ speakers, and two types of learners (children, adults), allowing for a comparison of different types of learners (first vs. second language, L1 vs. L2).

**Table 1:** Overview of participants.

	Mean age (year;month)	Age range (year; month)
**Learners**		
4-year-olds (*n* = 11; 6 f^1^)	4;7	4;1–5;4
6-year-olds (*n* = 11; 9 f)	6;8	6–7;8
9-year-olds (*n* = 11; 7 f)	9;2	8–10;9
Adult learners of L2 French (*n* = 11; 9 f)^2^	20	19–22
**Competent speakers**		
Adult Italian L1 (*n* = 11; 7 f)	22	19–31
Adult Dutch L1 (*n* = 11; 9 f)^2^	20	19–22

*^1^ f, female.*

*^2^ These are the same individuals.*

Thirty-three Italian children were recruited in Naples (*n* = 26) and Rome (*n* = 7). The 11 Italian adults were university students recruited in Naples at the Università degli Studi di Napoli “L’Orientale”. The 11 Dutch adults were recruited at Radboud University, Nijmegen, Netherlands. They participated twice, speaking L1 Dutch on one occasion, and L2 French on the other. At the time of recording they had studied French as a foreign language for a minimum of 4 years, and had never lived in a French-speaking country. In some cases, 3 years had lapsed between their last contact with the language and the time of testing. They were all at a low to intermediate proficiency level. All participants signed a consent form; parents signed consent forms for the children.

#### Materials

All participants retold cartoon stories. Two different cartoons were used as stimuli. The Italian participants (children and adults) were shown a video entitled *Pingu’s family celebrates Christmas* (The Pygos Group, 1992), an episode lasting 90 s. The Dutch participants (native speakers and learners) were shown a printed wordless cartoon featuring three gnomes trying to solve a problem (cf. Gullberg, 2006). Since narrative content and structure is irrelevant to the analyses in this study, the use of different cartoons to elicit narrative production was deemed to be unproblematic.

#### Procedure

The Italian participants were presented with the cartoon on a laptop that was removed after viewing. Children were recorded in a familiar setting, either their home or at school. They retold the story to a familiar adult (a friend of the family or their teacher). The adult, who had also seen the cartoon, was instructed not to interrupt the child during the retelling, not to suggest parts of the story (even when the child missed them), but to provide feedback showing interest and participation to the interaction (i.e., *ah, uhu, I see, how nice*). The Italian adults were recorded at university. Two participants were involved in each session: one person was asked to watch the cartoon and then to retell it to a friend who had not seen it. In order to make the Italian adult narratives comparable with those produced by the children, the listener was instructed to only listen to the story and to avoid interrupting the narrator, or to ask questions at the end of the story.

The Dutch participants were recorded at the Max Planck Institute for Psycholinguistics, Nijmegen, Netherlands, on two different occasions approximately a week apart: once in Dutch (the L1) and once in French (the L2). The order of the language/sessions was counterbalanced. The story was told to a confederate native speaker of the relevant language (Dutch for the L1 sessions, and French for the L2 sessions) who had not seen the cartoon. The interlocutor was instructed to ask clarification questions and provide feedback to create as naturalistic a session as possible.

#### Data Treatment and Coding

Data was transcribed and coded by frame-by-frame analysis of digital video in the annotation software ELAN (Wittenburg et al., 2006).

##### Speech

The retellings were transcribed using standard Dutch, French, and Italian orthography by native speakers. For the analyses presented here, all the L1 narratives (Dutch adults, Italian children and adults) were transcribed and analyzed in full (mean duration 2 min). Because the L2 narratives were considerably longer (mean duration 8 min), a selection was made of 2 min from the middle of the L2 recordings for transcription and analysis (see **Table 2**).

**Table 2:** Overview of duration of retellings.

	Mean duration retellings (min:sec)	Analyzed
**Learners**		
4-year-olds (*n* = 11)	02:12	02:12
6-year-olds (*n* = 11)	02:33	02:33
9-year-olds (*n* = 11)	02:25	02:25
Adult learners of L2 French (*n* = 11)	08:23	02:00
**Competent speakers**		
Adult Italian L1 (*n* = 11)	01:51	01:51
Adult Dutch L1 (*n* = 11)	03:01	03:01

Speech was coded as fluent when no disfluency markers were present, or as disfluent when one of the following disfluency markers was present (boldface = disfluency marker):

• Filled pauses (*les deux* deux ***eh*** *nains* ‘the two uh dwarfs’, D07L2);
• Unfilled pauses, minimum duration 200 ms, transcribed with (.) [*mettevano l’uovo* ***(.)*** *sopra* ‘they put the egg (.) on the top,’ ItCh12];
• Interruptions transcribed with apostrophe (*juste une* ***esc’*** *escalier* ‘just one flight of st’ stairs,’ D21L2);
• Lengthenings, transcribed with colon (*alla fine* ***esce:*** *l’albero di Natale*; in the end it comes: the Christmas tree,’ ItCh24);
• Repetitions (*una palla* ***di di di*** *neve* ‘a ball of snow,’ ItCh16);
• Combinations of these categories with at least two different kinds of disfluencies appearing in immediate sequence [*il a une* ***eh (.) eh*** *image* ‘he has a eh (.) eh image,’ D01L2].

Importantly, only intra-clausal occurrences of disfluency were considered. That is, phenomena occurring at clause boundaries (as in example 1) or following discourse markers (2) were excluded.

(1) *i regali che hanno fatto ai gentori* ***(.)*** *nella terza scena troviamo che* (ItAd17) ‘the presents that they had made for the parents (.) in the third scene we find that’
(2) *allora* ***(.)*** *ë vabbè l’inizio* (ItCh12) ‘well (.) uh well the beginning’

This selection was made to avoid over-estimating the amount of disfluencies. It is well-known that pauses often occur at clause- or utterance initial boundaries, and it is suggested that this is a consequence of the planning of the next clause (Maclay and Osgood, 1959; Hawkins, 1972, etc.). Moreover, it is also suggested that gestures are more likely to occur within than between clauses (cf. Beattie and Butterworth, 1979; McNeill, 1992, p. 94). In an examination of claims concerning speech and gestures in disfluency, instances of intra-clausal problems therefore seems like a better test bed where speech production has been launched and gestures are more likely to occur.

Twenty cases of repetition were excluded from analysis, since there were too few instances to perform further analysis. This procedure left 1,351 disfluencies for analysis. **Tables 3A,B** provide an overview of the aggregated and relative frequency distribution of fluent and disfluent stretches of speech across the groups, and the frequency of each of the disfluency markers, respectively.

**Table 3A:** Number and mean proportion of fluent and disfluent stretches of speech across groups.

Learners	# Fluent stretches	# Disfluent stretches	*M* % disfluent stretches (*SD*)
4-year-olds	308	85	0.21 (0.08)
6-year-olds	495	155	0.23 (0.05)
9-year-olds	537	221	0.30 (0.06)
Adult learners of L2 French	471	395	0.46 (0.08)
**Competent speakers**			
Adult Italian L1	719	285	0.28 (0.08)
Adult Dutch L1	603	210	0.26 (0.05)

**Table 3B:** Number of types of disfluencies across groups.

	Types of disfluencies				
Learners	UP	FP	I	L	C
4-year-olds	39	4	20	11	11
6-year-olds	17	16	48	38	36
9-year-olds	32	23	57	75	34
Adult learners of L2 French	110	118	38	32	97
**Competent speakers**					
Adult Italian L1	10	32	18	204	21
Adult Dutch L1	48	100	4	16	42

*UP, unfilled pause; FP, filled pause; I, interruption; L, lengthening; C, combination.*

##### Gestures

**FIGURE 1:**
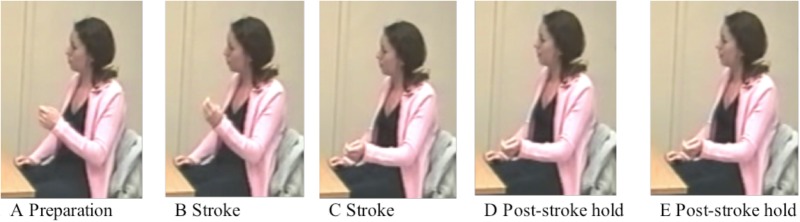
Example of gesture phases including ongoing stroke and post-stroke hold. **(A)** Preparation. **(B)** Stroke. **(C)** Stroke. **(D)** Post-stroke hold. **(E)** Post-stroke hold.

The gesture coding took the speech analysis as its departure point. First, for each fluent and disfluent stretch of speech, we coded for the presence or absence of a gesture. Second, gestures occurring with disfluent speech were further coded for their structural properties, that is, whether they were ongoing strokes or holds. Gestures were coded as ongoing when the stroke (i.e., the most effortful part of the gestural movement where the spatial excursion of the limb reaches its apex, cf. Kendon, 1980; McNeill, 1992; Seyfeddinipur, 2006) was being performed (**Figures 1B,C**). Gestures were coded as holds when there was a momentary suspension of movement, whether an interrupted or held preparation, or a post-stroke hold (**Figures 1D,E**; Kita et al., 1998). A total of 2,306 ongoing strokes, and 670 holds were identified. To give an overview of gestural activity in the data, we also computed mean gesture rate by word for each group, by dividing the total number of words (excluding interrupted words in disfluencies) with the total number of ongoing strokes per individual. We then computed the mean rate across each group. **Table 4** summarizes the distribution of ongoing strokes and mean gesture rate across groups to illustrate the properties of the sample.

**Table 4:** Frequency of gesture strokes and mean gesture rate/word across the groups.

	# Gesture strokes	Mean gesture rate/word (SD)
**Learners**		
4-year-olds	142	0.11 (0.06)
6-year-olds	325	0.13 (0.06)
9-year-olds	408	0.14 (0.05)
Adult learners of L2 French	392	0.29 (0.07)
**Competent speakers**		
Adult Italian L1	692	0.24 (0.07)
Adult Dutch L1	347	0.09 (0.03)
Total	2,306	

**FIGURE 2:**
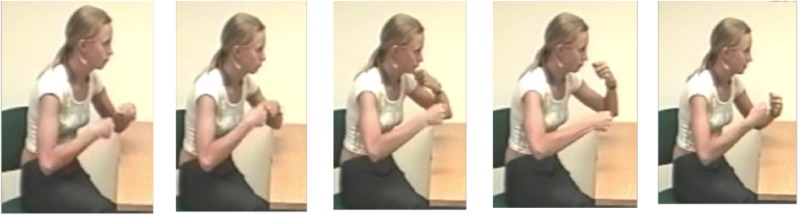
Example of a referential gesture depicting fist fighting.

**FIGURE 3:**
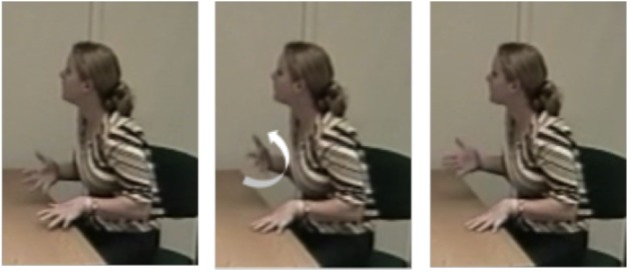
Example of a pragmatic gesture.

Third, we coded all ongoing strokes (both in fluent and disfluent speech) for function. Following Kendon (2004), we distinguished between referential and pragmatic functions. Gestures with a referential function (example in **Figure 2**) express semantic content through the depiction of referential properties (e.g., size, shape, and action) or indexical properties (deictic gestures and pointing). Gestures with a pragmatic function (example in **Figure 3**), in contrast, convey part of “an utterance’s meaning that [is] not part of its referential meaning or propositional content” (Kendon, 2004, p. 158). In other words, pragmatic gestures do not express referential content but rather function like speech acts by commenting on the speaker’s spoken production. For this coding, we excluded those gestures that could not be determined as having either a referential or pragmatic function (*n* = 35 or 8% of the total number of gestures).

Finally, a new coder coded 10% of the data across all groups. We computed interrater reliability measures (Cohen’s kappa, cf. Hallgren, 2012) for the identification of disfluencies, and gestures, the coding of gestures as ongoing vs. holds, and gesture function as referential or pragmatic (**Table 5**).

**Table 5:** Interrater reliability measures, Cohen’s kappa.

Group	Data	Observations	Raters	Kappa
Italian_L1	Speech disfluency	263	2	0.936
Italian_L1	Gesture	357	2	0.955
Italian_L1	Ongoing stroke/hold	267	2	0.867
Italian_L1	Gesture function	223	2	0.992
Dutch_L1	Speech disfluency	82	2	0.868
Dutch_L1	Gesture	90	2	0.937
Dutch_L1	Ongoing stroke/hold	37	2	0.874
Dutch_L1	Gesture function	35	2	0.968
Adult_L2	Speech disfluency	59	2	0.984
Adult_L2	Gesture	118	2	0.920
Adult_L2	Ongoing stroke/hold	116	2	0.987
Adult_L2	Gesture function	72	2	0.975

#### Analyses

For all analyses, we make (a) a crosslinguistic comparison of competent adult native speakers of Dutch and Italian; (b) a developmental comparison of three Italian child groups and adult Italian speakers; (c) a developmental comparison between competent adult native speakers of Dutch and adult Dutch L2 learners of French.

For the statistical analyses we used the glmerMod package in R, version 0.98.953 (R Core Team, 2014) to perform Generalized Linear Mixed-effects Models (GLMMs) with random intercepts for participants and items (Baayen, 2008; Baayen et al., 2008). Models were fit using maximum likelihood (Laplace approximation) [‘glmerMod’], binomial family (logit), since the dependent variable outcome throughout was binary. All analyses were run on raw numbers, but for ease of exposition figures show mean proportions.

## Results

### Gestures With Disfluent vs. Fluent Speech

**FIGURE 4:**
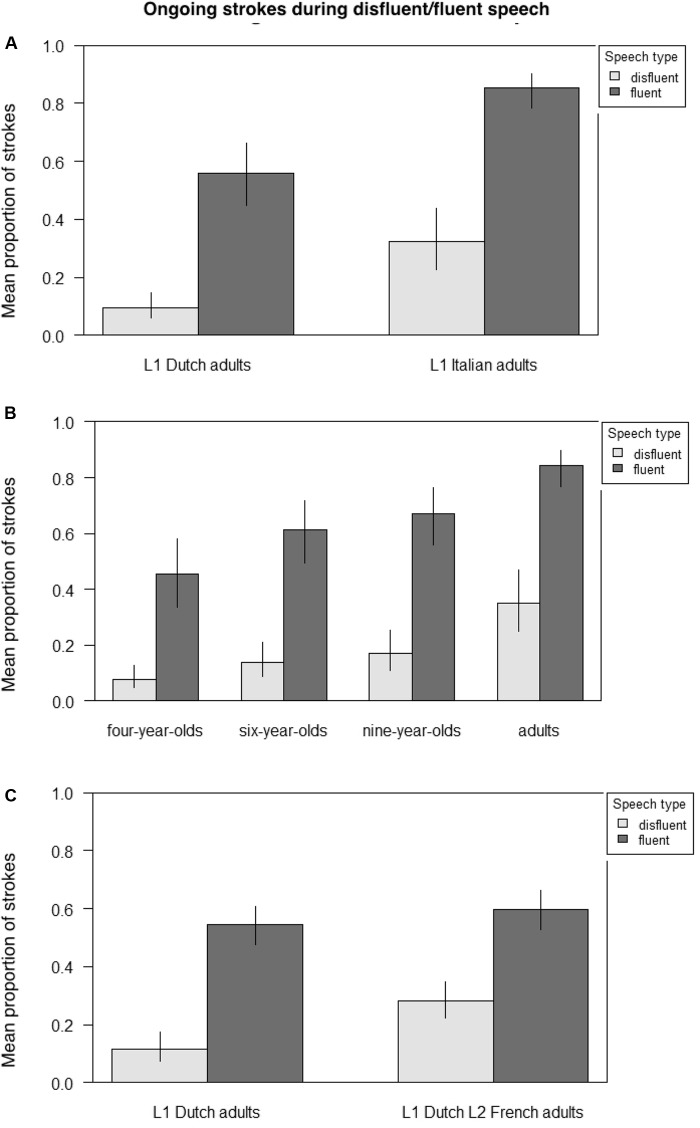
Mean proportion of ongoing strokes during disfluent/fluent speech across groups. **(A)** Adult native Dutch vs. Italian speakers. **(B)** Italian children aged 4, 6, and 9 vs. Italian adult speakers. **(C)** Adult native Dutch speakers vs. adult Dutch learners of L2 French.

**Figure 4** presents the mean proportion of ongoing strokes occurring with disfluent and fluent speech, respectively, comparing adult native Dutch and Italian speakers (**Figure 4A**), Italian 4-, 6-, and 9-year-olds and adult Italian speakers (4B), and adult native Dutch speakers and adult Dutch learners of L2 French (4C). **Table 6** presents the output from three GLMMs on the likelihood of gestures occurring with disfluent speech across groups, again, first examining adult native Dutch and Italian speakers; then Italian 4-, 6-, and 9-year-olds and adult Italian speakers; and finally, adult native Dutch speakers and adult Dutch learners of L2 French. Participants and items were always random factors, and group (Dutch/Italian; 4-/6-/9-year-old/adult Italian; L1/L2) and speech (disfluent/fluent) fixed main effects.

**Table 6:** Summary of Generalized Linear Mixed Models testing whether ongoing strokes occur with disfluent or fluent speech across groups.

	Est.	*SE*	*z*
**Adult Dutch/Adult Italian** (gesture ∼ group+speech+1(1 | participant)		
Intercept	-2.2550	0.2595	-8.690^∗∗∗^
groupItalianAdu	1.5190	0.3246	4.679^∗∗∗^
speechFluent	2.4910	0.1456	17.114^∗∗∗^
**Italian 4-/6-/9-year-olds/adults** (gesture ∼ group+speech+1(1 | participant)		
Intercept	-2.4717	0.2741	-9.017^∗∗∗^
groupItalian6ys	0.6368	0.3514	1.812
groupItalian9ys	0.8850	0.3499	2.529^∗^
groupItalianAdu	1.8585	0.3513	5.291^∗∗∗^
speechFluent	2.2942	0.1133	20.253^∗∗∗^
**Adult Dutch L1 vs. Adult L2 French** (gesture ∼ group^∗^speech+1(1 | participant)^†^		
Intercept	-2.0253	0.2408	-8.412^∗∗∗^
groupL2	1.0865	0.2430	4.471^∗∗∗^
speechFluent	2.1997	0.2313	9.512^∗∗∗^
groupL2:speechFluent	-0.8697	0.2738	-3.176^∗∗^

*p-values: ^∗∗∗^0.001, ^∗∗^0.01, ^∗^ 0.05.*

^†^*The model with the interaction term better explained the data and was therefore selected, χ^*2*^(1) = 10.802, p < 0.01.*

The results indicate that in all groups there was a main effect of speech type such that gestures were significantly more likely to occur with fluent than disfluent speech (adult Dutch/adult Italian, Est. = 2.491, *z* = 17.114, *p* < 0.001; Italian 4-/6-/9-year-olds/adults, Est. = 2.2942, *z* = 20.253, *p* < 0.001; and L1 Dutch/L2 French, Est. = 2.1997, *z* = 9.512, *p* < 0.001). In addition, the results reveal a shift over the course of child development, with Italian adults (Est. = 1.8585, *z* = 5.291, *p* < 0.001) and 9-year-olds (Est. = 0.885, *z* = 2.539, *p* < 0.05) differing from 4-year-olds who do not differ from 6-year-olds. Furthermore, for L2 speakers there is an interaction with speech type such that L2 speakers are significantly more likely than L1 speakers to produce gestures with disfluent speech (Est. = -0.8697, *z* = -3.176, *p* < 0.01).

The following examples illustrate the main pattern of absence of gestures during disfluencies. We follow Kendon (2004) in transcribing gestures: | = gesture phrase/unit boundaries; ∼∼ = preparation phase; ^∗∗^ = stroke; underlined = hold; -.- = recovery.

(3) adult Dutch native speaker D25L1
  en t’ derdremannetje die gaat er dus vandoormet ehm (.) de ladder
  |^∗∗∗∗∗∗∗∗∗∗∗∗∗∗∗∗∗∗∗∗∗∗∗∗∗^|^∗∗∗∗∗∗∗∗∗∗^|
  ‘and the third little man he just goes ahead with uh’

In (3) a Dutch native speaker says *en t’ derdre mannetje die gaat er dus vandoor met* ‘and the third little man he just goes ahead with’ producing two gestures. The first is a referential gesture where both hands have a tight grip handshape moving rightward, as if holding something and moving it. The second gesture is a pragmatic gesture where the both hands are twisted at the wrist to reveal palms up. When she then becomes disfluent, starting with a filled pause followed by a long silence, she drops both hands to the lap.

(4) adult Italian native speaker (ItAd05)
  il padre fuori l’igloo che: che: appunto addobba
  |^∗∗∗∗∗∗^|^∗∗∗∗∗∗∗∗∗∗^|
  ‘the father outside the igloo that: that: in fact decorate’

In (4) an Italian native speaker says *il padre fuori l’igloo* ‘the father outside the igloo’ and produces two gestures. The first is a pragmatic gesture (the index and thumb held together to form a ring). The second is a referential gesture performed with an open hand palm facing leftward that is moved laterally to the right side to indicate the outside. He then becomes disfluent and drops his hands to the lap.

(5) Italian child learner (ItCh12)
  invece al pappà un fiocchetto poi eh al ai al al: mh: al bimbo
  |∼∼^∗∗∗∗∗∗∗∗∗∗∗^-.-|
  ‘instead to the father a bow then eh to the to the to the to the: mh: to the child’

In (5), during the fluent part of speech, an Italian child produces a gesture representing the bow tie bringing both hands to the neck and outlining the shape of a bow tie. During the disfluent stretch she drops her hands to the lap.

(6) adult L2 learner of French (D25L2)
  et une (.) structure avec eh
  |*∼-.-*|      |*∼^∗∗∗∗∗∗^^∗∗∗∗∗^-.-*|
  ‘and a (.) structure with uh’

In (6), an adult L2 speaker launches a gesture preparation (cf. **Figure 1A**) as she says *une* ‘a,’ but then becomes disfluent and abandons the gesture immediately. Following this, during an exceptionally long unfilled pause (4 s 242 ms), she does nothing. Only when speech resumes with *structure* does she produce a gesture with a referential function, outlining a big triangle. The gesture goes into a hold as she says *avec* ‘with,’ and as she becomes disfluent again with a filled pause, she drops her hands and abandons the gesture.

### Ongoing Strokes vs. Holds During Disfluent Speech

**FIGURE 5:**
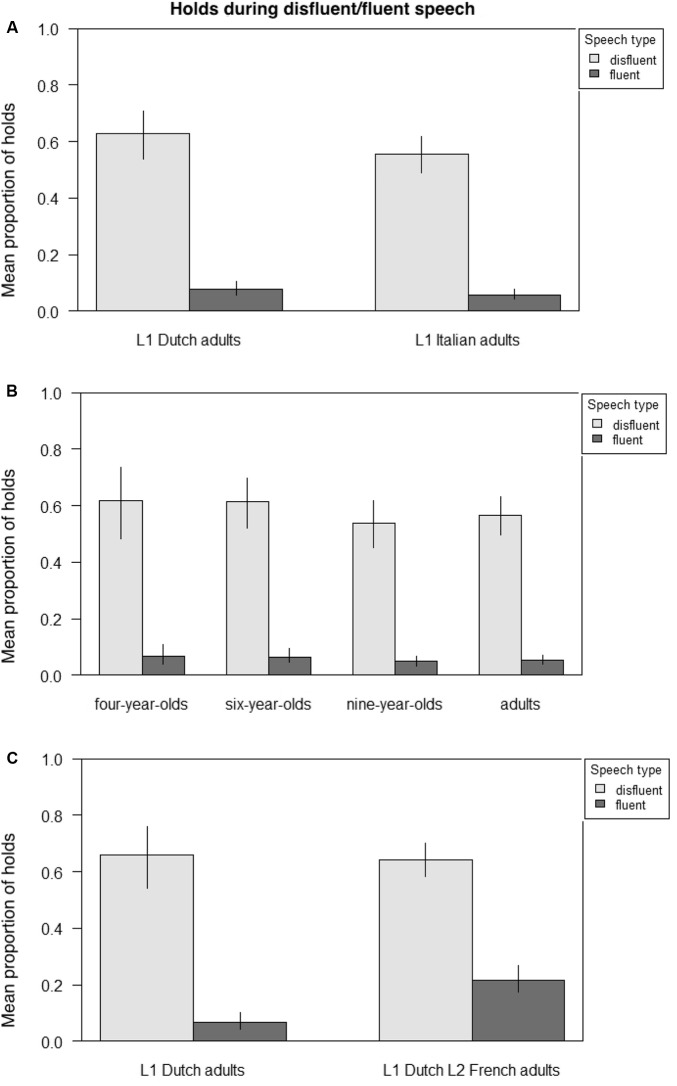
Mean proportion of gestural holds during disfluent/fluent speech across groups. **(A)** Adult native Dutch vs. Italian speakers. **(B)** Italian children aged 4, 6, and 9 vs. Italian adult speakers. **(C)** Adult native Dutch speakers vs. adult Dutch learners of L2 French.

**Figure 5** presents the mean proportion of holds across fluent and disfluent stretches of speech, respectively, comparing adult native Dutch and Italian speakers (**Figure 5A**), Italian 4-, 6-, and 9-year-olds and adult Italian speakers (5B), and adult native Dutch speakers and adult Dutch learners of L2 French (5C). **Table 7** presents the output from three GLMMs on the likelihood of holds occurring with disfluent speech across groups, again, first examining adult native Dutch and Italian speakers; then Italian 4-, 6-, and 9-year-olds and adult Italian speakers; and finally, adult native Dutch speakers and adult Dutch learners of L2 French. Participants and items were always random factors, and group (Dutch/Italian; 4-/6-/9-year-old/adult Italian; L1/L2) and speech (disfluent/fluent) fixed main effects.

**Table 7:** Summary of Generalized Linear Mixed Models testing whether gestural holds occur mostly with disfluent vs. fluent speech across groups.

	Est.	*SE*	*z*
**Adult Dutch/Adult Italian** (gesture2 ∼ group+speech2+1(1 | participant)		
Intercept	-0.5206	0.1877	-2.774^∗∗^
groupItalianAdu	0.2993	0.1919	1.559
speechFluent	3.0070	0.1815	16.570^∗∗∗^
**Italian 4-/6-/9-year-olds/adults** (gesture2 ∼ group+speech2+1(1 | participant)		
Intercept	-0.4770	0.2820	-1.692
groupItalian6ys	0.0140	0.3194	0.044
groupItalian9ys	0.3314	0.3127	1.060
groupItalianAdu	0.2154	0.2959	0.728
speechFluent	3.1174	0.1542	20.211^∗∗∗^
**Adult Dutch L1 vs. Adult L2 French** (gesture2 ∼ group^∗^speech+1(1 | participant)†		
Intercept	-0.6598	0.2542	-2.569^∗∗^
groupL2	0.0735	0.2742	0.268
speechFluent	3.2821	0.3262	10.062^∗∗∗^
groupL2:speechFluent	-1.4160	0.3699	-3.828^∗∗∗^

*p-values: ^∗∗∗^0.001, ^∗∗^0.01, ^∗^ 0.05.*

*^†^The model with the interaction term better explained the data and was therefore selected, χ^2^(1) = 15.519, p < 0.001.*

The results indicate that in all groups there was a main effect of speech type such that holds were significantly more likely to occur with disfluent than fluent speech (adult Dutch/adult Italian, (Est. = 3.007, *z* = 16.570, *p* < 0.001; Italian 4-/6-/9-year-olds/adults, Est. = 3.1174, *z* = 20.211, *p* < 0.001; and L1 Dutch/L2 French, Est. = 3.2821, *z* = 10.062, *p* < 0.001). There were no differences between the native speakers of Dutch and Italian, and no developmental effects in the child-adult comparison. However, for L2 speakers there was an interaction with speech type such that L2 speakers were significantly more likely than L1 speakers to produce holds with fluent speech (Est. = -1.4160, *z* = -3.828, *p* < 0.001).

In the interest of space, we provide only two examples from learners to illustrate the occurrence of holds during disfluencies.

(7) Child learner (ItCh12)
  vabbé l’inizio l: lasciamolo stare
  |∼∼∼∼∼∼^∗∗∗∗∗∗∗∗∗∗∗∗^-.-|
  ‘well the beginning l: let’s drop it’

In (7) an Italian 6-year-old prepares a gesture during the fluent stretch *l’inizio* ‘the beginning.’ She then becomes disfluent lengthening the consonant *l:* and at the same time suspends the gesture preparation going into a hold. When speech is resumed, the gesture is resumed and completed. She produces a referential gesture with the right hand open with palm facing downward moving laterally as if moving something aside.

(8) adult L2 learner of French (D17L2)
  le trois persons eh can eh (.) hu ehm
  |*∼∼∼∼∼^∗∗∗∗∗∗∗^^∗∗∗∗∗∗∗^-.-*|
  ‘the three persons eh can eh (.) *hu ehm*’

In (8), an L2 speaker produces a gesture with a referential function during the fluent stretch of L2 French, *le trois persons*, ‘the three persons,’ with both hands moving in a semi-circular movement as if grouping the three people. During the first filled pause (*eh*) the gestural movement goes into a hold and the speaker suspends her two hands. The hold continues during the subsequent disfluency until she abandons it, dropping her hands during the lengthy unfilled pause.

### Gesture Functions in Disfluent Speech

**FIGURE 6:**
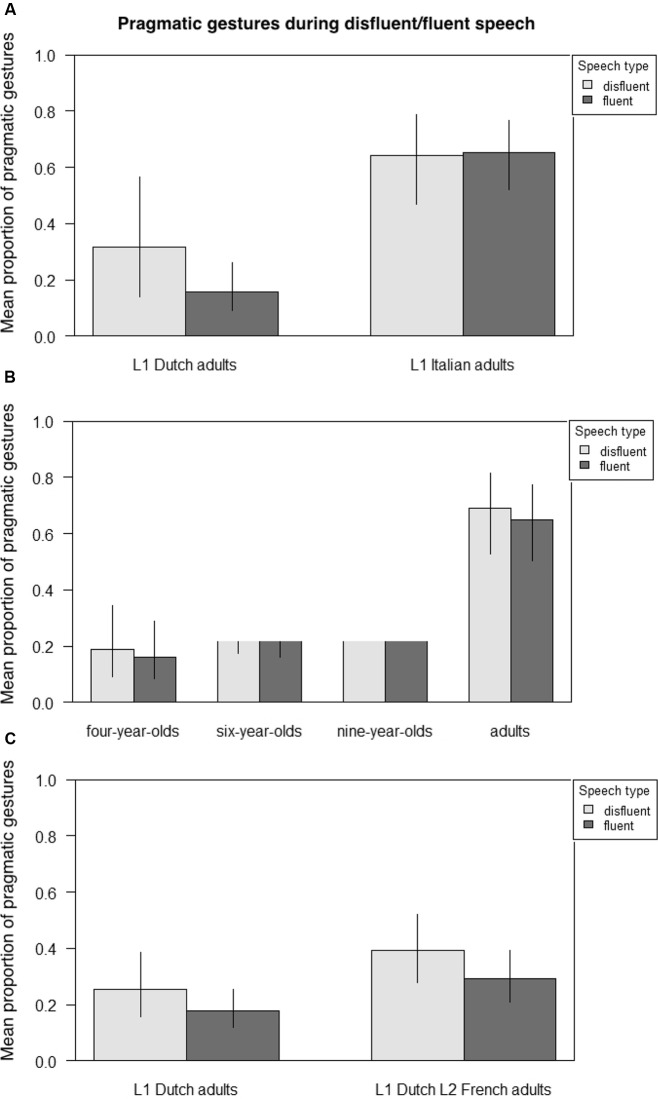
Mean proportion of pragmatic gestures during disfluent/fluent speech across groups. **(A)** Adult native Dutch vs. Italian speakers. **(B)** Italian children aged four, six and nine vs. Italian adult speakers. **(C)** Adult native Dutch speakers vs. adult Dutch learners of L2 French.

**Figure 6** presents the mean proportion of gestures with a pragmatic function across fluent and disfluent stretches of speech, respectively, comparing adult native Dutch and Italian speakers (**Figure 6A**), Italian 4-, 6-, and 9-year-olds and adult Italian speakers (6B), and adult native Dutch speakers and adult Dutch learners of L2 French (6C). **Table 8** presents the output from three GLMMs on the likelihood of pragmatic gestures occurring with disfluent speech across groups, again, first examining adult native Dutch and Italian speakers; then Italian 4-, 6-, and 9-year-olds and adult Italian speakers; and finally, adult native Dutch speakers in L1 and in L2 French. Participants and items were always random factors, and group (Dutch/Italian; 4-/6-/9-year-old/adult Italian; L1/L2) and speech (disfluent/fluent) fixed main effects.

**Table 8:** Summary of Generalized Linear Mixed Models testing whether pragmatic gestures occur mostly with disfluent vs. fluent speech across groups.

	Est.	*SE*	*z*
**Adult Dutch/Adult Italian** (gestfunction ∼ group+speech+1(1 | participant)		
Intercept	1.4438	0.3754	3.846^∗∗∗^
groupItalianAdu	-2.1988	0.4179	-5.261^∗∗∗^
speechFluent	0.1424	0.2363	0.603
**Italian 4-/6-/9-year-olds/adults** (gestfunction ∼ group+speech+1(1 | participant)		
Intercept	1.4698	0.4204	3.496^∗∗∗^
groupItalian6ys	-0.6383	0.5025	-1.270
groupItalian9ys	-1.3441	0.4953	-2.714^∗∗^
groupItalianAdu	-2.2660	0.4927	-4.599^∗∗∗^
speechFluent	0.1763	0.1859	0.949
**Adult Dutch L1 vs. Adult L2 French** (gestfunction ∼ group+speech+1(1 | participant)		
Intercept	1.0786	0.3109	3.469^∗∗∗^
groupL2	-0.6455	0.1948	-3.314^∗∗∗^
speechFluent	0.4498	0.2309	1.948

*p-values:^∗∗∗^0.001, ^∗∗^0.01, ^∗^ 0.05.*

The results indicate that in no group were pragmatic gestures more likely to occur with disfluent than fluent speech despite numerical trends in some groups. However, there was a crosslinguistic difference in that Italian speakers were more likely to produce pragmatic gestures with fluent speech than adult Dutch speakers (Est. = -2.1988, *z* = -5.261, *p* < 0.001). There was also a developmental effect in that Italian 9-year-olds (Est. = -1.3441, *z* = -2.714, *p* < 0.01) and adults (Est. = -4.266, *z* = -4.600, *p* < 0.001) were more likely to produce pragmatic gestures with fluent speech than 4- and 6-year-olds, who did not differ. Finally, adult L2 speakers were significantly more likely to produce pragmatic gestures with fluent L2 speech than L1 speech (Est. = -1.4160, *z* = -3.828, *p* < 0.001).

Examples (8) and (9) illustrate the occurrence of pragmatic gestures during disfluencies.

(9) Italian child learner (ItCh31)
  con matterello stava: (.)     stendendo la sfoglia per fare dei biscotti
         |*^∗∗^-.-*| |*^∗∗∗∗∗∗∗∗∗∗∗∗∗∗∗∗^*|
  ‘with the rolling pin was: (.) stretching out the pastry to make cookies’

In (9), an Italian 9-year-old hesitates and produces a gesture with a pragmatic function during the unfilled pause (.) with the right open hand rotated up and down twice. Once speech resumes, he continues to produce a referential gesture that represents the stretching out of the pastry with both hands.

(10) adult L2 learner of French (D21L2) < > = whispering
  ilest eh (.)          <putting>          ehm (.)     le maisonest
  |*^∗∗^*|*^∗∗^*|                          |^∗^*^∗^*|*^∗^*|        |     |*∼^∗∗∗∗∗∗^-.-*|
  ‘he is eh (.) <putting>ehm (.) the house is’

In (10), an L2 speaker produces a string of gestures with pragmatic functions during a long disfluent stretch, tapping her fingers with both hands on the table. These gestures are accompanied by averted gaze and a thinking face (cf. Goodwin and Goodwin, 1986; Gullberg, 2011). When she resumes speech saying *le maison* ‘the house,’ she simultaneously produces a gesture with a referential function, fingers tracing a square.

A final example (10) illustrates how an onstroking stroke with a referential function is produced during a disfluency by a L2 speaker (L2 = L2 speaker; NS = native speaker interlocutor).

(11) adult L2 learner of French (D07L2)
  L2: *ils sont (.) très ehm (.)*
                |*^∗∗∗^*
  NS: en colère
  *-.-.-.-*|
  LS: en colère et (.)
  |*∼^∗∗∗^^∗∗^*
  NS: ils se battent
  *^∗∗∗∗∗∗∗∗∗∗∗∗^-.-*|
  L2: oui oui
  L2: ‘they are (.) very uhm (.)
  NS: angry
  L2: angry and (.)
  NS: they fight
  L2: yes yes’

In the sequence in (11), after the L2 speaker initiates a fluent stretch, *ils sont très* ‘they are very,’ she becomes disfluent. In the second unfilled pause, she produces a gesture with a referential function representing the act of fighting with both fists moving around each other in a circle (cf. **Figure 2**). She shifts her gaze to the native interlocutor who offers a first solution, *en colère* ‘angry’ while the learner drops her hands. The L2 speaker repeats this phrase but is not satisfied, so she repeats the gesture in a third unfilled pause, again with gaze shifted to the native speaker. The learner’s gesture has gone into a hold and is held while the native speaker suggests *ils se battent* ‘they fight.’ The learner accepts this suggestion, drops her hands, and confirms, *oui oui* ‘yes yes,’ nodding. The referential ‘fighting gesture’ is thus used to elicit the lexical item from the interlocutor (cf. Gullberg, 1998, 2011).

## Discussion

This study examined the putative compensatory role of gestures by investigating their distribution, temporal, and functional properties relative to speech disfluencies in speakers of two different languages (Dutch and Italian), and with different degrees of linguistic expertise (child and adult language learners). The key findings can be summarized in four points. First, in all groups, speakers’ gesture production differs in fluent and disfluent stretches of speech, such that gestures overwhelmingly occur with fluent speech. Adult L2 speakers are more likely than anyone else to gesture also during disfluent speech. Second, in all groups gestures tend to be held during disfluent speech, not to be ongoing strokes. Third, the small number of ongoing gestures during disfluency display both pragmatic and referential functions. Adult L2 learners are more likely than anyone else to produce referential gestures during disfluency. Fourth, there are no crosslinguistic differences in gestural behavior during disfluencies. We only find a crosslinguistic difference in the production of pragmatic gestures during *fluent* stretches, with Italian adults producing more such gestures than Dutch adults and Italian children.

The overwhelming tendency for gestures to occur with fluent rather than disfluent speech does not support the first prediction by the Lexical Retrieval Hypothesis to the effect that, if gestures facilitate lexical retrieval, they should occur more frequently during speech disfluencies. Instead, the results suggest a very tight link between fluent speech and gesture production, supporting the notion that speech and gesture form an integrated or co-orchestrated system in speech production (e.g., McNeill, 1992; Clark, 1996; Kendon, 2004). The strikingly similar patterns found across speakers of different languages and across competent and learning language users alike support this notion quite forcefully.

The finding that any gestural activity found during speech disfluencies is mostly held or suspended in all groups similarly further reinforces the view of an integrated speech-gesture system. All speakers, children and adults, competent or learners, either interrupt an ongoing gesture when speech is interrupted (i.e., they stop or hold the preparation) or they freeze it (i.e., produce a post-stroke hold). That is, when speech stops, so does gesture. This finding is in line with and extends previous studies (e.g., Mayberry and Jaques, 2000; Seyfeddinipur and Kita, 2001; Yasinnik et al., 2005; Esposito and Marinaro, 2007), and provides supplementary evidence that holds or gesture suspensions tend to coincide with disfluency markers. It is also in line with McNeill’s suggestion of parallel break-downs (McNeill, 1985). These speaker-directed perspectives are complemented by findings on the functions of holds in interaction, which are relevant since the narratives analyzed here are interactive. For example, in seminal work Duncan (1972) showed that holds and ‘relaxation’ of tensed hands consistently occurred at the ends of turns in conversation thus signaling the end of a turn. When they linger after the turn, they have often been treated as cues to elicit a response from the interlocutor (Bavelas, 1994; Sikveland and Ogden, 2012; Cibulka, 2016, inter al.). Park-Doob (2010, p. 1) demonstrates that holds can “support continued expressiveness and interpretability,” that is they can indicate that the concept presented through the gesture is still active, thus allowing an interlocutor to draw information from a suspended gesture. Similarly, Cibulka (2016) reports that holds can be deliberately inserted in repair sequences to indicate that an entire utterance is momentarily suspended. Such functional analyses of holds in interaction are not in contradiction to the current findings concerning the speech production process. Instead, they provide a window on the multi-functionality of gestures in general and suspensions/holds in particular, whereby both speech and gesture production processes are subject to multiple influences in interaction (cf. Kendon, 2004).

Turning to gestural functions during disfluency, all groups produced not only referential but also pragmatic gestures in the small number of ongoing strokes found during disfluencies. Again, this result does not support the second prediction by the Lexical Retrieval Hypothesis, according to which we should expect referential gestures during disfluencies activating lexical items. As in the examples provided, the pragmatic gestures performed during disfluencies are not related to lexical content but rather to aspects of difficult interaction arising from the disfluencies both in adults and children (cf. Graziano, 2014a,b for similar findings on children). These gestures, often performed with a repeated oscillation of the open hand through wrist rotation or by tapping the fingers on a surface, provide a metalinguistic comment on the communication breakdowns, signaling that there is a problem in the speech production or that the speaker is engaging in a word search. Stam and Tellier (2017) classify word searching gestures as production oriented. This certainly tallies with these findings. However, although these gestures clearly indicate a production difficulty, they equally clearly have the potential to serve an interactive function (cf. Bavelas et al., 1992), indicating, for example, that the speaker is holding the floor. The averted gaze and the ‘thinking face’ (Goodwin and Goodwin, 1986) that often accompanies these gestures, suggest a strong floor-holding component.

Learners, both children and adults, overall revealed the same patterns as competent speakers, and there were no crosslinguistic differences in disfluencies. These findings highlight that the integrated behavior is pervasive. That said, the adult L2 speakers differed most from other groups both in speech and gesture. Although they overall pattern in the same way as the other groups, L2 speakers are more likely than native speakers to produce (ongoing and referential) gestures with disfluent speech. Although this result seems to support the predictions by the Lexical Retrieval Hypothesis, it is important to qualify the finding. First, it is not the dominant pattern even for L2 speakers. Second, ongoing strokes in disfluency have both pragmatic and referential functions. The pragmatic functions do not relate to lexical content, so cannot support lexical retrieval. Third, and most importantly, when referential gestures are produced during disfluencies, they tend to occur in specific contexts, illustrated by example (11). Here the L2 speaker seems to produce referential gestures strategically to elicit lexical help from the interlocutor – not from herself. In performing the ‘fighting’ gesture (cf. **Figure 2**) in silence, the L2 speaker certainly represents the concept she has trouble expressing, but she also uses the referential dimension of the gesture in combination with the direct gaze to the interlocutor with a pragmatic aim, namely to request help from the interlocutor, who does indeed provide a linguistic label for the gesture. Such sequences are relatively common in face-to-face interaction between L2 and native speakers (cf. Gullberg, 1998, 2011). There is further support for the crucial interactive aspect of such behavior. Holler et al. (2013) have shown that the communicative situation affects the rate of referential gestures in disfluency. During non-fluent speech, native speakers tend to produce more referential gestures during tip-of-the-tongue states when facing interlocutors than when they cannot see them or when they speak to a recorder. Overall, such patterns of production of referential gestures in disfluencies support Kendon’s (2004) claim that gestures, depending on the context, can have multiple functions at the same time; namely, in this case, referential and pragmatic/interactive. Obviously, this is not to say that referential gestures are never produced instead of lexical items or never ease their production. But we do claim that this cannot be considered the main function of gestures, not even for L2 speakers.

A further result from the L2 speakers is that they rather surprisingly produce more holds with *fluent* speech than anyone else. One possible reason for this is that the L2 speakers under study really are beginners with low levels of proficiency. They are therefore highly disfluent. In fact, they are so disfluent that their ‘fluent’ stretches of speech tend to be very short, consisting only of one or two words, and to be ‘inserted’ between disfluencies, rather than the other way around. Examples (6) and (9) illustrate this quite clearly. In such situations, suspensions or holds from a disfluency can ‘spill over’ to the fluent part of an utterance. On the whole, then, L2 speakers display more of everything than the other groups – they are more disfluent than any other group, but their predominant pattern of no gesture or hold in disfluency is the same as for all. They also produce more ongoing strokes with referential functions in disfluencies than anyone else. This is presumably a reflection of the fact that they may have a communicative intention ready in their first language which they cannot express lexically in the second language. Their referential gesture can thus reflect a lexical notion in the L1 when they decide to use the gesture to elicit help from an interlocutor. But if the word is not known in the L2, then no amount of gesturing can activate it.

It is important to acknowledge that the Lexical Retrieval Hypothesis makes predictions specifically concerning lexical difficulties in the domain of spatial language, assuming that referential gestures will crossmodally prime spatial vocabulary. The current analyses have not taken the specifics of lexical information into account, but rather applied a global analysis to all intra-clausal disfluencies. Partly, this is because we have conducted a corpus analysis on naturalistically occurring disfluencies in narrative corpora. In such contexts, it is not always easy to know whether the sought word is spatial or not, nor whether the resolution is even related to the original lexical problem (cf. Seyfeddinipur, 2006 for similar comments). However, it seems unlikely that the overwhelmingly clear patterns found in the four corpora analyzed would change for spatial language specifically. That said, an experimental study could be undertaken inducing disfluency and targeting specific semantic domains to see whether the type of analysis performed here would yield similar results. This would also address other drawbacks with the corpus analysis such as differing elicitation methods across corpora both as regards stimulus materials (printed/video) and common ground (whether interlocutors also saw the stimuli or not). Both differences may have affected overall gesture rate, for example, and although gesture rate was not of interest *per se* in this study, it may have influenced the sample size.

The current results provide no or little support for the Lexical Retrieval Hypothesis proposing that ongoing referential gestures in disfluencies help speech production. But what about the ongoing pragmatic, or rather non-referential, gestures? Following other authors, we have suggested that these gestures comment on the break-downs in interactive settings. However, suggestions are found in the literature to the effect that non-referential gestures may serve a speaker-directed purpose, helping to stimulate and focus attention thus keeping “communicative speech “on course”” (e.g., Grand et al., 1977, p. 499; cf. Stam and Tellier, 2017). Admittedly, many findings are linked to the study of populations with psychiatric conditions, but they open potential new avenues of exploration.

## Conclusion

Overall, the results from the present study suggest a very tight link between fluent speech and gesture production, providing strong support for the notion that speech and gestures form a tightly integrated or co-orchestrated system, with similar properties across languages and speakers’ skills. The findings constitute an important challenge for gesture theories assuming a mainly (lexical) compensatory role for (referential) gestures. Moreover, the observation that gestures that do accompany disfluencies have both pragmatic and referential functions raises further important challenges for gesture theories which have hitherto been based on subsets of gestures (referential) and solely on adult, competent, fluent speakers. The findings are also challenging for theories of language acquisition that tend to view gestures mainly as a (lexical) crutch. Perhaps most importantly, the findings are a challenge for mono-modal theories of language who look only to (written forms of) spoken or signed language, ignoring gestures as irrelevant. The data strongly suggest that when speech stops, so does gesture across languages, across age, and across types of learners. Speech disfluency is generally mirrored by gesture disfluency. To us, this suggests that gesture production is part and parcel of language production, and therefore worthy of linguistic theorizing more broadly.

## Ethics Statement

This study was carried out in accordance with the recommendations of the Regional Ethical Review Board at Lund University with written informed consent from all subjects (note that the data were collected while the authors were employed in the Netherlands and Italy, but that the Swedish board has reviewed the protocol). All subjects gave written informed consent in accordance with the Declaration of Helsinki.

## Author Contributions

All authors listed have made a substantial, direct and intellectual contribution to the work, and approved it for publication.

## Conflict of Interest Statement

The authors declare that the research was conducted in the absence of any commercial or financial relationships that could be construed as a potential conflict of interest.

## Footnotes

1 Referential gestures are also known in the literature as representational, sometimes further labeled iconic/metaphoric gestures (e.g., McNeill, 1992). We will use the term referential gesture, following Kendon, 2004.

2 A related suggestion is that gestures may relieve cognitive load although this is not specifically related to language (e.g., Goldin-Meadow et al., 2001; Hostetter and Sullivan, 2011; Cook et al., 2012).
